# Effector Mechanisms of Neutrophils within the Innate Immune System in Response to *Mycobacterium tuberculosis* Infection

**DOI:** 10.3390/jcm6020015

**Published:** 2017-02-07

**Authors:** Eric Warren, Garrett Teskey, Vishwanath Venketaraman

**Affiliations:** 1 Department of Basic Medical Sciences, College of Osteopathic Medicine of the Pacific, Western University of Health Sciences, 309 East Second Street, Pomona, CA 91766-1854, USA; ewarren@westernu.edu; 2 Graduate College of Biomedical Sciences, Western University of Health Sciences, Pomona, CA 91766-1854, USA; gteskey@westernu.edu

**Keywords:** Tuberculosis, TB, *Mycobacterium tuberculosis*, *M. tb*, polymorphonuclear leukocytes, PMN, Neutrophils, Innate immunity, NETs, Cytokines, Phagocytosis-induced cell death

## Abstract

Neutrophils have a significant yet controversial role in the innate immune response to *Mycobacterium tuberculosis* (*M. tb*) infection, which is not yet fully understood. In addition to neutrophils’ well-known effector mechanisms, they may also help control infection of *M. tb* through the formation of neutrophil extracellular traps (NETs), which are thought to further promote the killing of *M. tb* by resident alveolar macrophages. Cytokines such as IFN-γ have now been shown to serve an immunomodulatory role in neutrophil functioning in conjunction to its pro-inflammatory function. Additionally, the unique transcriptional changes of neutrophils may be used to differentiate between infection with *M. tb* and other bacterial and chronic rheumatological diseases such as Systemic Lupus Erythematosus. Adversely, during the innate immune response to *M. tb*, inappropriate phagocytosis of spent neutrophils can result in nonspecific damage to host cells due to necrotic lysis. Furthermore, some individuals have been shown to be more genetically susceptible to tuberculosis (TB) due to a “Trojan Horse” phenomenon whereby neutrophils block the ability of resident macrophages to kill *M. tb*. Despite these aforementioned negative consequences, through the scope of this review we will provide evidence to support the idea that neutrophils, while sometimes damaging, can also be an important component in warding off *M. tb* infection. This is exemplified in immunocompromised individuals, such as those with human immunodeficiency virus (HIV) infection or Type 2 diabetes mellitus. These individuals are at an increased risk of developing tuberculosis (TB) due to a diminished innate immune response associated with decreased levels of glutathione. Consequently, there has been a worldwide effort to limit and contain *M. tb* infection through the use of antibiotics and vaccinations. However, due to several significant limitations, the current bacille Calmette-Guerin vaccine (BCG, vaccine against TB) does not meet the criteria for universal utilization for all ages and populations across the globe. New research involving neutrophils has yielded a new vaccine called *M. smegmatis*-Ag85C-MPT51-HspX (mc^2^-CMX) that has been shown to elicit a humoral and cellular response against *M. tb* in mice that is superior to the BCG vaccine.

## 1. Introduction

Tuberculosis (TB) is an infectious disease that caused 10.4 million illnesses and 1.8 million deaths worldwide in 2015 [1]. In addition, the World Health Organization (WHO) estimates that currently one third of the world’s population is latently infected with *M. tb* [1]. TB is caused by the bacterium *Mycobacterium tuberculosis* (*M. tb*) and is transmitted from person to person via aerosol droplets. Although TB primarily affects the lung parenchyma, it may also impact bone, the central nervous system, and other organ systems [2]. A vaccine against TB called bacille Calmette-Guerin (BCG) is currently available and is commonly utilized in foreign countries with a high prevalence of TB, in order to prevent tuberculous meningitis in children. However, this vaccine is generally not recommended in the United States due to its limited efficacy in adults and because it may result in a false positive reaction to the Tuberculin Skin Test (TST) [3]. The treatment of active TB disease includes several first-line antibiotics which include: Isoniazid, Rifampin, Ethambutol, and Pyrazinamide. However, the treatment of TB disease can become more complicated due to the development of drug-resistant and multidrug-resistant strains of *M. tb* [3]. In addition to the various treatment regimens for TB, the immune system plays an integral role in defense against *M. tb* infection [4,5]. Innate immune cells such as polymorphonuclear leukocytes (PMN), or neutrophils have been shown to be circumstantially unfavorable during *M. tb* infection [6]. However, they have also been shown to help mediate an early inflammatory response that is critical for controlling *M. tb* infection [7,8,9]. Historically, neutrophils have been underappreciated phagocytes. The focus of early immunologists was aimed at understanding mononuclear phagocytes such as monocytes and macrophages, despite the fact that neutrophils constitute approximately 50% to 80% of all circulating white blood cells and contribute to the innate immune response via phagocytosis of invading bacteria, degranulation, and subsequent secretion of cytokines such as tumor-necrosis factor-alpha (TNF-α) and interleukin 1 (IL-1) [7,10,11,12,13,14,15,16]. Neutrophils also secrete reactive oxygen species (ROS), elastase, collagenase, and myeloperoxidase, factors that have been shown to damage both invading mycobacterial cells and host cells in a nonselective manner [9,16,17].

## 2. Neutrophil Extracellular Traps and Its Effector Functions

**Figure 1:**
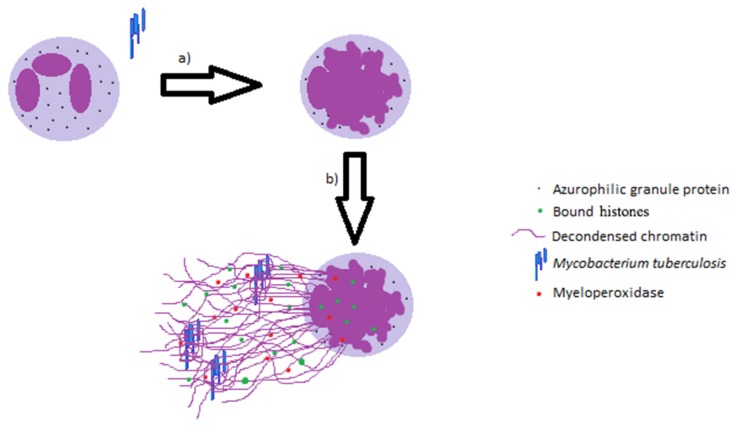
Formation of neutrophil extracellular traps (NETs) during the innate immune response to *M. tb*. (**a**) Structural alterations of the nuclear shape with chromatin decondensation; (**b**) release of the intracellular structures and net exposure.

While some studies state that neutrophils are unable to kill virulent *M. tb*, others explicitly contradict these findings [18,19,20]. Regardless, these important innate immune cells can help contain the infection and contribute to early granuloma formation [21]. Neutrophils are also known to help modulate the effector mechanisms of resident alveolar macrophages (AM). There are several ways in which neutrophils augment the killing of *M. tb* by macrophages. The formation of neutrophil extracellular traps (NETs), which contain decondensed chromatin, bound histones, azurophilic granule proteins, myeloperoxidase, and cytosolic proteins, (Figure 1) has been shown to trap the invading mycobacteria and therefore prevent the spread of *M. tb* to other organs. The exuding of these structures by neutrophils is thought to not only limit microbial spread and dissemination, but also to enhance the effective concentrations of released microbicidal agents [21,22,23,24]. Additionally, the *M. tb*-induced NETs have been shown to sequester toxic contents to protect surrounding tissues from becoming damaged [22]. Macrophages may also acquire and utilize the antimicrobial peptides derived from phagocytosed NETs [22]. Lastly, the release of heat shock protein 72 (Hsp72) by NETs in response to *M. tb* triggers a pro-inflammatory response in resident alveolar macrophages, thereby causing the release of IL-6, TNF-α, and IL-1β [22].

Figure 1 illustrates the role of neutrophil extracellular traps in contributing towards innate immune responses against *M. tb* infection.

## 3. Cytokines Modulating the Functions of Neutrophils

An effective host innate immune response against *M. tb* infection is based not only upon successful cell-mediated killing of *M. tb*, but also on the efficient regulation of innate immune cells, often mediated by cytokines [20,25,26]. Neutrophils in circulation can be directed into infected tissues, such as TB ridden lung parenchyma, by cytokines leading to their activation and engulfment of the pathogen. Neutrophils are also an important source of specific cytokines that can help aid in early recruitment and activation of other innate immune cells, contributing to cellular immunity against mycobacterium infection [27]. For example, neutrophils produce the cytokine tumor-necrosis factor-alpha (TNF-α), which stimulates dendritic cells and macrophage differentiation and activation, a necessary response in TB infection [14,15,28]. Additionally, TNF-α is known to have a protective role against TB infection and play a major role in host defense against these intracellular pathogens, aiding in phagocytosis, the activation of T-cells, as well as granuloma formation [20,25,26,29,30,31,32,33,34]. In response to *M. tb*, neutrophils will also release the chemokine IL-8, which has a central role in leukocyte recruitment to areas of granuloma formation, as well as respiratory burst in neutrophils [35,36,37,38,39]. Therefore, the available data suggest that *M. tb* virulence is inversely proportional to the neutrophilic secretion of most cytokines and chemokines [40,41]. Conversely however, the recruitment of neutrophils to infected lung parenchyma is negatively influenced by the cytokine interferon- gamma (IFN-γ). While IFN-γ is pro-inflammatory, it also been shown to inhibit the accumulation of neutrophils, and impair neutrophil survival, which can be beneficial to the host if too many neutrophils have been recruited to the infection site [42]. IFN-γ has been shown to prevent neutrophil accumulation by inhibiting the production of IL-17, which is an important signal in neutrophil recruitment. Reduced levels of IL-17 will result in the reduction of the neutrophil hematopoietic factor, and granulocyte-colony stimulating factor (G-CSF) [42].

## 4. Neutrophils Apoptosis and Phagocytosis Induced Cell Death

Studies of *M. tb* have identified an unequivocal relationship between the virulence of the bacteria and the initiation of apoptosis. *M. tb* survival and propagation is favored by the inhibition of phagocyte apoptosis [16,43,44]. Additionally, it has been proposed that neutrophils may accelerate immune priming for *M. tb* by initiating an infection induced apoptosis in order to pass the phagocytized bacteria to migratory dendritic cells to expedite their trafficking to lymph nodes [45]. Therefore, neutrophilic phagocytosis is an integral part of the effective innate immune response against invading *M. tb*. After phagocytosis of harmful bacteria, the timely removal of spent neutrophils is also of equal importance, due to their relatively short life span (approximately 5.4 days), and the potential for their cytotoxic molecules to be displaced within the surrounding tissues upon their death, known as necrotic lysis [46,47,48]. Therefore, a mechanism known as phagocytosis-induced cell death (PICD) is implemented to clear these spent neutrophils by efferocytosis [48]. In the process of PICD mediated efferocytosis, neutrophils will undergo specific cell surface changes such as the switching of phosphatidylserine, and oxidizing lipids, to their cell surface, marking them for recognition by surface receptors on macrophages [49]. PICD is initiated after phagocytosis of complement or antibody-coated particles as well as bacteria. Neutrophilic phagocytosis of various strains of bacteria including *M. tb*, *E. coli*, and *Neisseria gonorrhoeae* has been shown to significantly accelerate the rate of apoptosis of neutrophils and limit the chances of necrotic lysis [48,50]. This association whereby pathogenic bacteria are directly involved in the functioning of neutrophils is important for the induction of cell turnover, as well as in the regulation of the inflammatory response. The influence that pathogenic bacteria have on the survival of neutrophils undoubtedly affects the regulation of cell turnover [51]. However, more importantly, are the microorganism’s role in the promotion of PICD and the subsequent effect on the inflammatory response. Not only does the phagocytosis of invading microorganisms induce a neutrophil mediated pro-inflammatory response, but rapid initiation of apoptosis is also coupled with the downregulation of these neutrophil pro-inflammatory products [52,53,54,55,56].

## 5. Genotypic Changes Affecting Neutrophil Functions

Genetic variations in mice have been shown to influence the proficiency of the effector mechanisms of neutrophils in the innate immune response against *M. tb.* One study found that mice with the I/St genotype were more susceptible to *M. tb* than mice with the A/Sn genotype. The difference in susceptibility to *M. tb* between these two genetic variants largely consisted of differing neutrophilic functioning within the lungs of mice that were challenged with *M. tb*. Specifically, neutrophils from the I/St mice were more numerous, lived longer, and had a higher migration capacity than neutrophils from the A/Sn mice. It is hypothesized that the mycobacteria are temporarily hidden from activated alveolar macrophages, which are able to effectively kill *M. tb*. This “Trojan Horse” phenomenon may be due to the resistance of neutrophils to IFN-γ. Additionally, neutrophils from the more susceptible mice did not up-regulate the CD95 apoptotic receptor, which when ligated, resulted in Fas-induced cell death. Therefore, the I/St mice were unable to effectively control the cell turnover of neutrophils in the lung, which in turn caused an excess amount of neutrophil inflammation, lung pathology, and increased severity of TB disease [57].

## 6. Neutrophil Transcriptional Changes in Chronic Diseases

TB disease displays a similar profile of innate immune cells to that of other infectious and inflammatory diseases such as Group A Streptococcus or Staphylococcus infection, as well as from Systemic Lupus Erythematosus (SLE). One study identified a specific 86-transcript neutrophil driven blood signature unique to *M. tb* infection. This signature is characterized by an IFN-inducible gene profile, which consists of both IFN-γ and Type I IFN-αβ signaling. The blood signatures of Streptococcus and Staphylococcus infections showed an insignificant change in the IFN-inducible module, while the profiles of SLE and TB are characteristically more similar. However, TB disease may be distinguished from SLE due to its overall pattern of transcriptional changes, as well as its differing amplitude [58].

## 7. Neutrophils and Granulomatous Responses against *M. tb* Infection

Early granulomas are composed of inflammatory macrophages, neutrophils, and dendritic cells that gradually accumulate upon recruitment to the site of infection [59]. In the beginnings of an early granuloma, macrophages will engulf the mycobacteria and subsequently become infected. Of the two types of lesions caused by *M. tb*, proliferative and exudative, the former involves the formation of intragranulomatous necrosis. Whereas the proliferative type of lesion does not typically progress to active TB, the exudative form involves the infiltration of neutrophils. There are four factors that are associated with the ability of neutrophils to infiltrate *M. tb* lesions: the breathing amplitude for bacillary drainage (BAM), the encapsulation capacity of the intralobar septae, the tolerability of the intracellular bacillary load (BLTOL), and the ability to counterbalance the Th17 response during the immune phase [60]. The BLTOL is related to an increased number of bacilli per macrophage which is known as the multiplicity of infection (MOI). A high MOI promotes tissue necrosis and the subsequent attraction of neutrophils to the alveolar site of infection [61]. Once the neutrophils have been recruited to the infection site, it is thought that they will phagocytize the bacilli and subsequently kill it, independent of ROIs, within these lesions [20]. Additionally, neutrophils in human granulomas have now been shown to be significant contributors to granzyme B (grzB) expression [62]. GrzB acts on intracellular substrates including pro-caspase 3, which then contributes to pathogen clearance [63].

## 8. HIV Infection and Type 2 Diabetes Association with Neutrophil Immune Responses to *M. tb*

The effectiveness of neutrophils in the innate immune response to *M. tb* may be encumbered due to an immunocompromised state. For instance, increased neutrophil counts are common among patients with active TB and a poorly functioning acquired immune response [64]. Co-infection with HIV has been shown to compromise an early line of defense against mycobacteria as well as increase susceptibility to *M. tb* due to decreased levels of IFN-γ, as a consequence of diminished numbers of CD4 T cells [61,65]. To combat this, activation of antigen-specific CD4 T cells has been shown to be facilitated by neutrophils [45]. Therefore, in immunocompromised individuals with impaired adaptive immunity, the innate immune response must work harder in order to provide protection from this co-infection marked by an increase in innate response cell parameters [62,66]. Functional defects of neutrophils are also seen in HIV, *M. tb* co-infected individuals which may contribute to an impaired host defense against opportunistic pathogens [67]. A study of PMN function in TB patients reported that untreated TB patients had a lower ROI production then normal individuals, which was even more pronounced in advanced patients, and the ROI production was nearly absent in the HIV co-infected group [68]. Additionally, Type 2 diabetes mellitus is also associated with an increased susceptibility for the development of TB. The underlying mechanism for this increased risk similarly involves neutrophils [69]. One of the major metabolic products of gut bacterial fermentation is short-chain fatty acids (SCFAs). Increased levels of SCFAs, particularly butyrate, have been shown to cause decreased neutrophilic mycobacterial phagocytosis, as well as decreased neutrophilic production of superoxide, hydrogen peroxide, and hypochlorous acid [70]. Due to altered levels of SCFAs, Type 2 diabetes confers a threefold increased risk for the development of TB. Individuals with Type 2 diabetes mellitus and HIV infection also have compromised levels of glutathione (GSH) [61,71,72]. In the case of HIV infected individuals, this leads to an inappropriate immune response by promoting a CD4^+^ T-helper 2 (T_H_2) directed response, which results in cytokines that ineffectively defend against intracellular pathogens such as *M. tb*. Conversely, high levels of GSH result in the inhibition of growth of *M. tb* inside of neutrophils [64].

## 9. Future Directions: Developing Vaccines That Will Induce Neutrophil-Mediated Favorable Immune Responses against *M. tb* Infection

As previously stated, the BGC vaccine is commonly used to prevent TB meningitis in children in developing countries, but is often not used in the United States due to its limited efficacy in adults, and because it may result in a false positive TST reaction. Thus, it is of vital importance to create a vaccine that is effective for both children and adults. A newly formulated recombinant live vaccine vector called mc^2^-CMX has recently been demonstrated to induce both a humoral and cellular response in mice that were exposed to *M. tb* that is superior to BCG [73]. The enhanced immune response conferred by this vaccine is largely due to the generation of Th1- and Th17-specific responses by neutrophils [74]. Given the tremendous role of neutrophils in eliciting an enhanced immune response in mice vaccinated with mc^2^-CMX, we suspect that medicine would benefit greatly due to additional research directed towards the function of neutrophils in additional vaccines.

## 10. Concluding Remarks

We have reviewed the known beneficial mechanisms of action of neutrophils during *M. tb* infection to illuminate their essential role in innate immunity. In summary, the interaction between neutrophils and *M. tb* remains a challenging subject; however, there is evidence that they mediate a critical and often debated role in the control of *M. tb* infection. In addition to their microbicidal effects, the present results suggest that they may also exert several immunomodulating effects during tuberculosis. Their importance is not only directly involved in contributing to the body’s defense against *M. tb* infection, but the efficient regulation of neutrophils is paramount to avoid deleterious effects such as necrotic lysis and the “Trojan Horse” phenomenon. Although major milestones have been accomplished in the elucidation of neutrophils in the innate immune response to *M. tb*, additional research is needed in areas such as TB vaccine development, as well as the role of genetics and environmental factors in neutrophilic functioning.
